# Photodynamic Inactivation Using Natural Bioactive Compound Prevents and Disrupts the Biofilm Produced by *Staphylococcus saprophyticus*

**DOI:** 10.3390/molecules26164713

**Published:** 2021-08-04

**Authors:** Wei Yang, Ziyuan Wang, Qing Li, Yating Jia, Shuimiao Song, Zichu Ma, Jie Liu, Jing Wang

**Affiliations:** 1 China-Canada Joint Lab of Food Nutrition and Health (Beijing), Beijing Technology and Business University, Beijing 100048, China; yangwei811127@126.com (W.Y.); foodstudying@163.com (Q.L.); jytwell@163.com (Y.J.); songshuimiao@foxmail.com (S.S.); mazichu123@163.com (Z.M.); liu_jie@btbu.edu.cn (J.L.); 2 College of Basic Science, Tianjin Agricultural University, Tianjin 300392, China

**Keywords:** natural bioactive compound, photodynamic inactivation, *Staphylococcus saprophyticus*, biofilm

## Abstract

*Staphylococcus saprophyticus*, the food-borne bacteria present in dairy products, ready-to-eat food and environmental sources, has been reported with antibiotic resistance, raising concerns about food microbial safety. The antimicrobial resistance of *S. saprophyticus* requires the development of new strategies. Light- and photosensitizer-based antimicrobial photodynamic inactivation (PDI) is a promising approach to control microbial contamination, whereas there is limited information regarding the effectiveness of PDI on *S. saprophyticus* biofilm control. In this study, PDI mediated by natural bioactive compound (curcumin) associated with LED was evaluated for its potential to prevent and disrupt *S. saprophyticus* biofilms. Biofilms were treated with curcumin (50, 100, 200 µM) and LED fluence (4.32 J/cm^2^, 8.64 J/cm^2^, 17.28 J/cm^2^). Control groups included samples treated only with curcumin or light, and samples received neither curcumin nor light. The action was examined on biofilm mass, viability, cellular metabolic activity and cytoplasmic membrane integrity. PDI using curcumin associated with LED exhibited significant antibiofilm activities, inducing biofilm prevention and removal, metabolic inactivation, intracellular membrane damage and cell death. Likewise, scanning electronic microscopy observations demonstrated obvious structural injury and morphological alteration of *S. saprophyticus* biofilm after PDI application. In conclusion, curcumin is an effective photosensitizer for the photodynamic control of *S. saprophyticus* biofilm.

## 1. Introduction

*Staphylococcus saprophyticus* (*S.* *saprophyticus*) is an important food-borne Gram-positive bacteria, which is typically isolated from livestock industry and environmental sources, such as soil, air and water [1]. Moreover, *S. saprophyticus* is commonly found in the food processing industry, such as on the surfaces of working spaces including floors, drains and processing machines, and can survive after routine cleaning and disinfection due to biofilm formation [2]. As coagulase-negative staphylococci (CNS) strains, *S. saprophyticus* carries multiple enterotoxin genes and is able to produce a range of heat-enterotoxins, causing food intoxication and human illness [3]. It was reported that over 90% of *S. saprophyticus* isolated from ready-to-eat food carried multi-antibiotic resistance, posing severe threats to public health [4]. Moreover, *S. saprophyticus* is also a constituent of the genitourinary tract microbiota and can cause urinary tract infections (especially in young women) [5].

Biofilms are aggregates of microorganisms adherent to each other and/or to surfaces, surrounded by self-produced extracellular polymeric substances (EPSs), which helps bacteria survive in hostile environments [6]. A biofilm consists of microbial cells adherent to one another living within an organic polymer matrix that they produce and to a static interface (living or nonliving) [7,8]. In staphylococci species, the biofilm matrix is mainly composed of polysaccharide intercellular adhesin (PIA). However, for *S. saprophyticus*, surface-associated protein (Ssp) is the most important component of biofilm, presented in 98% of *S. saprophyticus* isolates [9]. Nowadays, these bacteria become a big challenge to human health due to the adaptation of strategies against antibiotic treatment like biofilm formation, efflux pump and secretion of various enzymes [10]. In order to control and eradicate bacterial biofilms, various physical and chemical strategies, including ultraviolet, flushing and sanitizers, have been applied, but only with limited effectiveness [11]. Consequently, alternative approaches able to inhibit or disperse biofilm production based on novel concept is in urgent need.

Photodynamic inactivation (PDI), combing a photosensitizer (PSs) with appropriate wavelength visible light in the presence of oxygen, has been regarded as promising for microbial decontamination [12]. Although the use of antibiotics has provided an option and been adopted for microbial control, the emergence of resistant bacteria has turned the antibiotic treatment of bacterial infections into a challenge [13,14]. The interest in PDI is due to the simple mechanism of action, as the exposure of photosensitizer to the specific light could induce the formation of reactive oxygen species (ROS), which are highly reactive to various biological molecules (DNA, proteins and lipids) and able to cause damage to many fundamental structures of the cell, resulting in cell death [15]. The antimicrobial ability of PDI to bacteria, virus, and fungi has been proven for various applications; however, the commonly used PSs are not suitable for food-related application. Currently, most PSs are ALA-based compounds and porphyrins, which are frequently used in medical application [16]. Curcumin, a yellow pigment obtained from the rhizome of *Curcuma longa*, is a natural bioactive compound and food-grade photosensitizer with a maximum absorption band at 430 nm, making it an alternative candidate in antibacterial agents in the food industry [17]. It has been reported to control the microbial load and extend the shelf life of fresh wet noodles without causing a negative impact on noodle quality [18]. At present, photosensitization with curcumin has been successfully tested on fungi and bacteria, such as *Candida albicans*, *Candida dubliniensis*, *Staphylococcus aureus*, *Staphylococcus epidermidis*, *Streptococcus mutans*, *Vibrio parahaemolyticus* and *Listeria monocytogenes* [19,20,21,22,23,24,25,26].

As little is known about the response of *S. saprophyticus* to photodynamic treatment, especially when a biofilm is present, the aim of our study was to verify the effectiveness of curcumin-mediated PDI against *S. saprophyticus* in its biofilm form under different conditions, including various doses of curcumin and light exposure. This is a pilot study for the assessment of natural bioactive component curcumin as a possible anti-biofilm agent for microbial biofilm control to ensure public health.

## 2. Results and Discussion

### 2.1. Effect of PDI on the Prevention of S. saprophyticus Biofilm Formation

**Figure 1:**
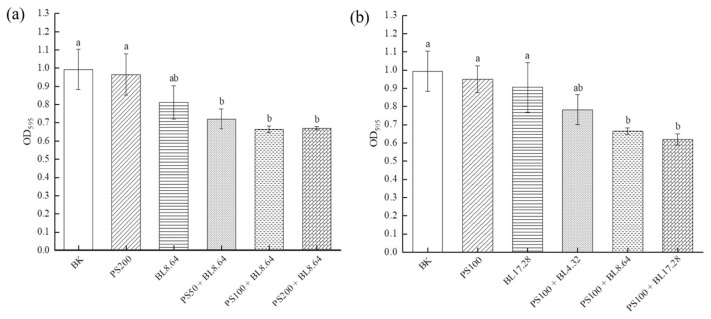
Inhibitory activity of PDI against *S. saprophyticus* biofilm formation under different CUR concentration (**a**) and light dose (**b**). BK: blank control; PS: photosensitizer treatment; BL: blue-light treatment; PS + BL: photosensitizer combined blue-light treatment. Values are presented as mean ± SD. Results marked with the same letters are not significantly different.

As one important specie of opportunistic coagulase-negative *Staphylococci*, the control and prevention of *S. saprophyticus* is important for reducing cross-contamination and maintaining microbial safety. To examine whether PDI prevented *S. saprophyticus* biofilm formation, the capacity for developing biofilm was investigated by crystal violet staining assay (Figure 1). The results indicated that the absorbance at OD_595_ in the control groups were higher than that of PDI groups, when treated with different concentrations of curcumin (50–200 μM) associated with an 8.64 J/cm^2^ dose (Figure 1a). Individual LED light or curcumin treatment did not cause significant reduction in mass production compared to blank control. The different concentrations of curcumin (50, 100, 200 μM) evaluated displayed no significant difference in antimicrobial photoinactivation, showing bacterial cells susceptible to PDI using the three concentrations. Additionally, as shown in Figure 1b, the OD_595_ value decreased significantly (*p* < 0.01) after 8.64 J/cm^2^ and 17.28 J/cm^2^ irradiation associated with 100 μM curcumin treatment, achieving a reduction of 33.1% and 37.6%, respectively. The findings showed that PDI using curcumin and blue LED exhibited good preventive effects on *S. saprophyticus* biofilm formation.

The results of this work corroborate with previous study that evaluated the effectiveness of PDI mediated by curcumin and blue light on *S. saprophyticus* in its planktonic form. It was observed that PDI using blue LED (4.32 J/cm^2^) associated with CUR (25 μM) caused significant reduction of *S. saprophyticus* (~5 log CFU/mL) in vitro, proving its potential in inhibiting the growth of planktonic cultures of *S. saprophyticus* [27]. As bacteria in the biofilm state exhibited higher tolerance to antimicrobial agents or treatment compared to the planktonic forms, higher concentrations of PS or dose of irradiation are required for effective biofilm inhibition. According to Martins et al., the biofilm cells of *S. saprophyticus* displayed a marked increase (>32 times) in minimum inhibitory concentration in biofilm (MICB) in comparison to the cells in planktonic condition, indicating that the biofilm was able to impair the penetration of antibiotics via extracellular matrix, which might act as a physical barrier for molecule diffusion and decrease the concentration available to the cells [3]. Another reason is that biofilm extracellular matrix is able to adsorb or interact with the antibiotics, in consequence, lowering the level of antibiotics available to the cells in the biofilm [28]. The enhanced resistance of biofilm against bacterial antibiotics might also be associated with persister cells, which are metabolically dormant, with an overexpression of drug efflux pumps, contributing to their higher tolerance to antimicrobial agents [29]. Moreover, it has been reported that bacterial biofilms would undergo a phenotypic change, such as increased thickness of cell wall, which renders them highly tolerant to antibiotics [30]. The resistance mechanisms might also include variations in membrane sterol composition, overexpression of efflux pumps and different developmental phases. In another investigation, PDI using curcumin (20, 40 or 80 μM) and blue light (455 ± 3 nm) at dose 5.28 J/cm^2^ was efficient to inactivate both methicillin-susceptible and -resistant *Staphylococcus aureus* biofilms, highlighting the potential of curcumin as an effective photosensitizer [31].

### 2.2. Effect of PDI on the Eradication of Pre-Established S. saprophyticus Biofilm (CFU Assay)

**Figure 2:**
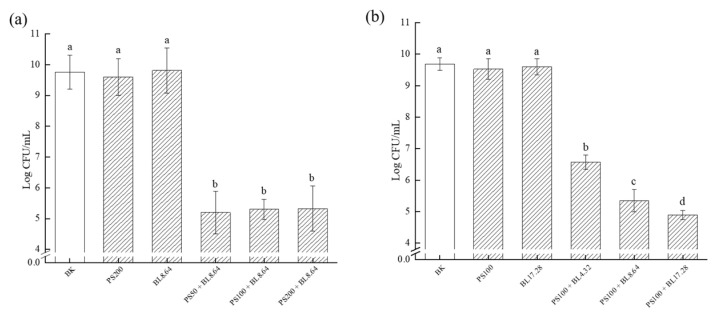
Viable bacterial counts of *S. saprophyticus* biofilm after PDI treatment under different curcumin concentration (**a**) and light dose (**b**). BK: blank control; PS: photosensitizer treatment; BL: blue-light treatment; PS + BL: photosensitizer combined blue-light treatment. Values are presented as mean ± SD. Different letters indicate significant difference between the test samples (*p* < 0.01).

Efficient antimicrobial approach should also have the ability to reduce the viability of existing biofilms, rather than only inhibiting biofilm formation. To investigate whether PDI was capable of disrupting and removing pre-formed *S. saprophyticus* biofilm, the effect of PDI-associating curcumin and blue light on biofilm sessile cells viability was assessed (Figure 2). No significant difference among the control groups (BK, PS and BL) was verified, suggesting that curcumin and light alone would not significantly reduce pre-formed biofilm cells viability. Compared with the control groups, notable reduction of *S. saprophyticus* viability was observed after all PDI treatments, achieving approximately 4.5 log CFU/mL reduction with different CUR concentrations (50, 100 and 200 μM) at 8.64 J/cm^2^ (Figure 2a). However, there was no statistical difference among the concentrations applied, which might occur due to the fact that the dose of 8.64 J/cm^2^ was probably insufficient for the complete photoactivation of all the PS molecules. Thus, the complete degradation of CUR probably did not occur, along with a limited formation of reactive oxygen species (ROS), resulting in a restricted phototoxic effect of curcumin. Therefore, compared with the lower concentration of CUR, PDI with higher concentrations were not able to cause more reduction in biofilm cell viability.

Additionally, the influence of light dose on the effectiveness of PDI on biofilm cells viability was evaluated. The result displayed an irradiation dose-dependent manner with the number of log CFU/mL reduction increasing along with the dosage of blue light (Figure 2b). PDI at light dose of 17.28 J/cm^2^ caused the highest reduction (4.8 log CFU/mL). A lower dose of 4.32 J/cm^2^ and 8.64 J/cm^2^ combined CUR treatment also induced marked reduction in the counts of bacteria on plates (3.12 log CFU/mL and 4.34 CFU/mL, respectively). Likewise, no significant difference was observed among all control groups. These findings corroborated the data from metabolic activity and CLSM observations. Similar results were reported by Teixeira et al., in that PDI mediated by curcumin (80 μM) and LED light (5.28 J/cm^2^) was able to cause approximately 3 log CFU/mL reduction of *Staphylococcus aureus* cell in biofilm [31]. These results were also agreed by Quishida et al., who evaluated the effectiveness of PDI using curcumin (80 μM and 100 μM) and LED (37.5 J/cm^2^) against 24 h biofilm of *Streptococcus mutans* and found that no significant difference in viability was verified between samples treated either with PS or light only and samples received neither PS nor LED light. Furthermore, there was no significant difference among PDI treatment groups when curcumin concentrations were compared [32].

### 2.3. Effect of PDI on Biofilm Metabolic Activity

**Figure 3:**
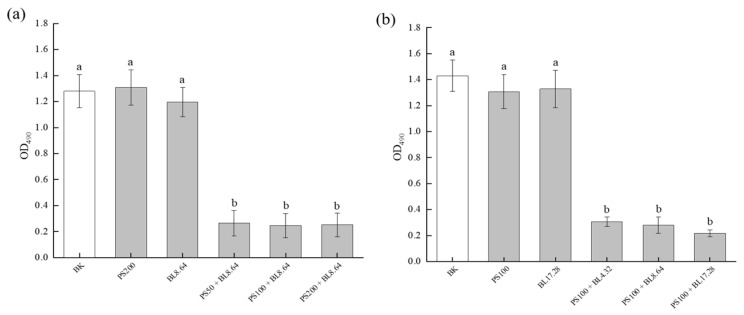
Effect of PDI on *S. saprophyticus* biofilm metabolic activity under different curcumin concentration (**a**) and light dose (**b**). BK: blank control; PS: photosensitizer treatment; BL: blue-light treatment; PS + BL: photosensitizer combined blue-light treatment. Values are presented as mean ± SD. Different letters indicate significant difference between the test samples (*p* < 0.01).

MTT assay was conducted to evaluate the effect of PDI on the metabolic activity of *S. saprophyticus* biofilms. As shown in Figure 3, biofilm cultures without PDI treatment maintained high metabolic activity, as ascertained by the high measured absorbance, and no significant difference was found among control groups treated only by photosensitizer (PS) or by blue light (BL) and the sample without light and PS treatment (BK). After PDI application, a significant difference (*p* < 0.01) in the absorbance values was observed in all treated groups with different curcumin concentrations (50 μM, 100 μM and 200 μM) compared with the controls (Figure 3a). The three concentrations of curcumin with blue LED light (8.64 J/cm^2^) were able to reduce biofilm cell metabolic activity. Likewise, compared with the control groups, PDI also resulted in a significant difference in the absorbance values at OD_490_ for all light doses. Although no significant difference was found among different light doses at the same PS concentration, the highest reduction was 84.76% for the PDI associated with the dosage of 17.28 J/cm^2^. Moreover, no significant difference was obtained when samples treated either with CUR (PS) or blue light (BL) only and samples with neither PS nor light (BK) treatment were compared (Figure 3b). These data indicate that *S. saprophyticus* biofilm was susceptible to the PDI, confirming the results observed in the CFU/mL test. The result was also in agreement with the finding by Sanitá et al. [24], who proved a significant decrease in *Candida dubliniensis* biofilm metabolic activity after PDI mediated by curcumin for all the concentrations applied.

### 2.4. Effect of PDI on Biofilm Membrane Integrity

**Figure 4:**
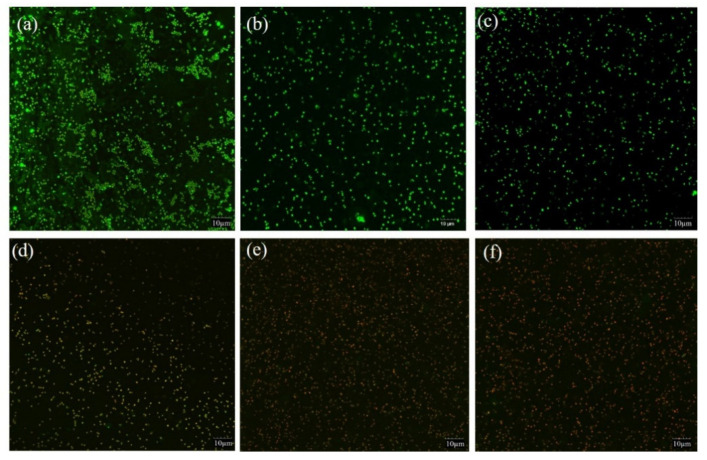
CLSM images of *S. saprophyticus* biofilm. (**a**) BK: blank control; (**b**) 100 μM CUR treatment only; (**c**) 17.28 J/cm^2^ irradiation treatment only; (**d**) PDI treatment with 100 μM CUR and 4.32 J/cm^2^ irradiation; (**e**) PDI treatment with 100 μM CUR and 8.64 J/cm^2^ irradiation; (**f**) PDI treatment with 100 μM CUR and 17.28 J/cm^2^ irradiation.

Biofilm cell membrane integrity was evaluated by CLSM based on PI penetrating (red fluorescence) into the cell, indicating damaged or death cell. The fluorescent dye SYTO 9 with green fluorescence indicated living cells with intact cell membrane. The changes of *S. saprophyticus* biofilm after PDI mediated by curcumin associated with blue light are shown in Figure 4. Compared with the controls (BK, PS and BL) (Figure 4a–c), there was a notable increase in the population of red cells after PDI application, demonstrating that photosensitization treatments were able to cause photodamage and compromise cell integrity effectively (Figure 4d–f). The data were in agreement with the results of biofilm cells viability and metabolic activity, suggesting that impairment of membranes could ultimately cause cell death. The mechanism might be ascribed to the production of ROS by PDI, targeting cellular biomolecules including nucleic acid, proteins and lipids and disrupting microbial cell membrane. Our result was in agreement with the other studies, in which the authors verified a notable increase in cells marked with red fluorescence after PDI by curcumin (80, 100, 120 μM) associated with light excitation on the biofilms of *Streptococcus mutans, Candida albicans* and *Candida glabrata* [32].

### 2.5. Morphological Alteration of S. saprophyticus Cells

**Figure 5:**
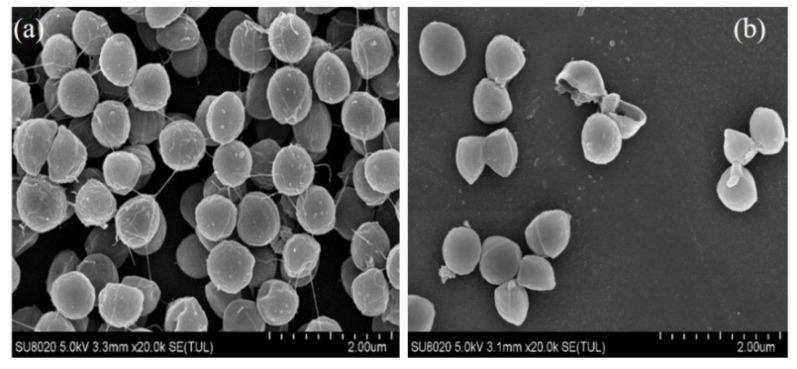
SEM images of *S. saprophyticus* biofilm. (**a**) Bacteria control group, lots of bacterial cells aggregated together and encased by extracellular polymeric substances. (**b**) Biofilm inhibition by PDI treatment, a small number of bacterial aggregates.

The morphological damage of PDI-treated *S. saprophyticus* mature biofilm was determined by SEM analysis (Figure 5). For the sample without PDI treatment (Figure 5a), biofilm cells exhibited uniform and dense structure, in addition to accumulated cells and abundant extracellular matrix. No significant alterations of the *S. saprophyticus* cells were observed. However, after PDI application, a reduced number of bacterial cell and deformed morphology was observed (Figure 5b). The distorted morphology of treated *S. saprophyticus* cells as well as leakages of intracellular components verified the effectiveness of PDI in eradicating pre-formed biofilms, which was in accordance with the result of biofilm sessile cells viability analysis.

## 3. Materials and Methods

### 3.1. Materials

Curcumin was obtained from Sigma-Aldrich (St. Louis, MO, USA), crystal violet was purchased from Solarbio (Beijing, China). MTT (3-[4,5-dimethylthiazol-2-yl]-2,5-diphenyltetrazolium bromide) was purchased from Beijing Biotopped Biotechnology Co., Ltd. (Beijing, China). Ethanol was obtained from Beijing Chemical Plant Co., Ltd. (Beijing, China). Acetic acid and methanol were obtained from Sinopharm group Co., Ltd. (Beijing, China). Tryptone Soy Agar (TSA) and Trypticase Soy Liquid Medium (TSB) were ordered from Beijing Aoboxing Biotechnology Co., Ltd. (Beijing, China). Glutaraldehyde was obtained from Tianjin Fuchen Chemical Reagent Co., Ltd. (Tianjin, China). DMSO, PBS and sodium chloride (NaCl) were supplied by Shanghai Macklin Biochemical Technology Co., Ltd. (Shanghai, China).

### 3.2. Bacterial Strain and Culture Conditions

*Staphylococcus saprophyticus* isolated from fresh noodle samples processed in our lab was used in the study. The glycerol stock culture was stored at −20 °C with 30% glycerol. Prior to bacterial viability analysis, the stock cell culture was thawed and cultured at 37 °C in TSB overnight with shaking. Then, *S. saprophyticus* was plated onto a TSA plate and incubated at 37 °C for 24 h. Then, bacterial single colony was inoculated into fresh TSB for logarithmic growth. The bacterial suspension was adjusted to OD_600nm_ 0.8 (~10^7^ CFU/mL) by using the microplate reader (Infinite F200 PRO, Tecan, Switzerland).

### 3.3. Crystal Violet (CV) Staining

Biofilm formation of *S. saprophyticus* was performed according to the reported method, and biofilm mass was quantified by crystal violet (CV) staining assay [33]. Briefly, 300 µL of bacterial suspension (10^7^ CFU/mL) and 900 µL of fresh TSB medium were added to sterile 48-well microtiter plates, incubated at 37 °C for 24 h. Then, the culture supernatant was carefully aspirated from the microtiter plates’ well, and the wells were washed thrice with sterile PBS (pH 7.4) to rinse non-adherent cells. Afterwards, 200 μL methanol (10%) were added to each well and maintained for 10 min for remaining bacteria fixation. Afterwards, methanol was removed and the fixed bacteria were stained with 100 μL CV dye (0.1% *v*/*v*) for 5 min. Sterile PBS was used to remove exceeding dye and rinse samples. Subsequently, acetic acid (1 mL, 33% *v*/*v*) was added to each well to dissolve the dye bound to the cells with careful shaking. To quantify biofilm mass, the solution (200 μL) was transferred to a microtiter plate and the absorbance at 595 nm was measured by a microplate reader (Infinite F200 PRO, Tecan, Switzerland). The experiment was conducted in triplicate.

### 3.4. PDI Treatment for Biofilm Prevention

Photodynamic inhibition experiments were performed by using a blue LED device (430–470 nm, Philips, Holland) [34]. Aliquots of 300 μL *S. saprophyticus* suspension (OD_600nm_ = 0.8) were inoculated into 900 μL of fresh TSB medium supplemented with different curcumin concentration solutions (50 µM, 100 µM and 200 µM). After dark incubation for 30 min, the *S. saprophyticus* suspensions were exposed to blue light of different doses (4.32 J/cm^2^, 8.64 J/cm^2^ and 17.28 J/cm^2^, respectively). Samples exposed to neither blue light nor CUR were set as blank control (BK); samples treated with blue light exposure alone were presented as illumination control (BL); samples treated with curcumin alone were presented as photosensitizer control (PS). After the PDI treatment, *S. saprophyticus* cultures were incubated at 37 °C for 24 h in 48-well microtiter plates for biofilm formation. The experiment was conducted in triplicate.

### 3.5. PDI Treatment for Biofilm Eradication

The eradication effects of curcumin-mediated PDI on pre-established biofilm were investigated according to a previously described method [35]. Prior to the treatment, 50 μL of *S. saprophyticus* suspension (OD_600nm_ = 0.8) were inoculated into 150 μL of fresh TSB medium in 96-well microtiter plates, and incubated at 37 °C statically for 24 h. The culture medium was carefully aspirated, and the wells were washed thrice with sterile PBS to remove non-adherent cells. Then, 200 μL of curcumin solutions with different concentrations (50 µM, 100 µM and 200 µM) were applied in each well and maintained for 30 min at 37 °C, followed by blue-light exposure with different doses (4.32 J/cm^2^, 8.64 J/cm^2^ and 17.28 J/cm^2^, respectively). Control groups including neither curcumin nor light exposure (BK), photosensitizer treatment alone (PS) and blue-light treatment alone (BL) were performed. The experiment was conducted in triplicate.

### 3.6. Quantification of Biofilm Culturable Cells

To assess the anti-biofilm efficacy of PDI, the biofilm was evaluated by the viability in plate medium after PDI treatment. The biofilms were rinsed with sterile PBS to remove weakly adherent cells, scrapped and resuspended in 200 μL of sterile PBS. The obtained suspension was moved to sterile microcentrifuge tubes and subjected to tenfold serial dilution in sterile PBS. Afterwards, aliquots of each dilution were plated onto TSA plates and incubated at 37 °C for 24 h. Then, the number of colony-forming units was counted and the results were represented in CFU/mL. All the procedures were applied three times.

### 3.7. MTT Assay

The metabolic activity of *S. saprophyticus* biofilm was evaluated by MTT assay according to Liu et al., with slight modifications [36]. After PDI treatments, 100 µL of MTT solution (0.5 mg/mL) were applied to each well containing biofilm samples, and incubated at 37 °C in darkness for 3 h. Afterwards, the reaction mixture was removed and 200 µL of DMSO were added to solubilize the formazan crystals. The reduced formazan product was determined by measuring the absorbance at 490 nm in a microtiter plate reader (Infinite F200 PRO, Tecan, Switzerland). The reduction of biofilm metabolic activity was calculated using the control group (cells incubated in the absence of CUR and without light exposure) as 100% of the metabolism of biofilms. All the procedures were applied three times.

### 3.8. CLSM Analysis

The membrane integrity of *S. saprophyticus* sessile cells after PDI treatment was evaluated by the Live/Dead BacLight Bacterial viability kit (Invitrogen, San Diego, CA, USA), observed using CLSM. Briefly, aliquots of 500 μL *S. saprophyticus* suspension (OD_600nm_ = 0.8) and 2 mL fresh TSB medium were added into each well, followed by incubation at 37 °C for 24 h. Afterwards, the supernatant was removed and the wells were rinsed twice with sterile PBS followed by PDI treatment as described above. The fluorescent dye (1 μL) containing SYTO-9 and propidium iodide (PI) were prepared according to the manufacturer’s instructions. The sessile cells were stained with the dye at room temperature for 15 min in darkness. Samples were observed by Confocal laser scanning microscopy (CLSM, FV3000, Olympus, Tokyo, Japan), in which the green fluorescence represented viable cells with intact cell membrane, while the red fluorescence represented damaged/dead ones. Excitation wavelengths of 488 nm and 561 nm were used for SYTO9 (480/500 nm) and PI (490/635 nm), respectively.

### 3.9. Scanning Electron Microscope (SEM) Analysis for Biofilm Morphology

To determine the morphological changes of PDI treatment, *S. saprophyticus* biofilm was observed by SEM, described by Garcez et al. with slight modification [37]. Briefly, aliquots of 500 μL *S. saprophyticus* suspension (OD_600nm_ = 0.8) and 2 mL fresh TSB medium were added to 6-well microtiter plates, followed by incubation for 24 h at 37 °C for biofilm formation. Afterwards, biofilms on the coverslips were washed twice with PBS followed by PDI treatment as described previously. Then, the biofilm was washed thrice with sterile PBS, fixed with 2.5% glutaraldehyde for 12 h at 4 °C. The fixed samples were passed through an increasing ethanol series (15, 30, 50, 70, 90, and 100%) for dehydration, dried, sputter-coated with gold, and visualized under a scanning electron microscope (SU8020, Hitachi, Tokyo, Japan).

### 3.10. Statistical Analysis

The experimental data were presented as mean ± SD. To determine significance, the results were analyzed by using SPSS statistical software (SPSS 19) and one-way ANOVA, and *p* < 0.01 was considered significant.

## 4. Conclusions

To the best of our knowledge, although the effect of curcumin-mediated PDI against other staphylococci (*S. aureus*) has been investigated, there is no report on its effect on *S. saprophyticus* biofilm formation and eradication. Due to the ability to produce enterotoxigenic, the attachment of *S. saprophyticus* to food-processing machines and contact surfaces could cause food safety issues, posing risks for the food industry. Antimicrobial properties of curcumin along with its negligible side effects on human health suggest it as a green and safe agent for microbial control. The current investigation proved that natural bioactive compound curcumin-mediated PDI was able to decrease the growth of *S. saprophyticus* and its capacity to form biofilms. Moreover, PDI was also able to disassemble the mature biofilm of *S. saprophyticus*, which diversified the application of bioactive compound in coping with bacteria biofilm-associated contaminations. Considering that infections involving biofilms create significant health and economic impacts, the PDI can be considered a promising approach to act synergistically for microbial control in the food industry.

## Data Availability

All data included in this study are available upon request by contact with the corresponding author.

## Sample Availability

Samples of the experiment are available upon request by contact with the corresponding author.
